# The Legality of Vitamin K Refusal in the United States

**DOI:** 10.7759/cureus.79368

**Published:** 2025-02-20

**Authors:** Avrohom Levy, Shira Nabatian

**Affiliations:** 1 Pediatrics, Jersey Shore University Medical Center, Neptune, USA; 2 Attorney, New York University, New York, USA

**Keywords:** law, medicolegal, neonatology, newborn care, pediatrics, public health, vaccine refusal, vitamin k, vitamin k deficiency bleeding, vitamin k prophylaxis

## Abstract

Antivaccine rhetoric has been a major topic of discussion in politics, the news, and on social media platforms. As social media use has become a mainstay of communication, it has become increasingly difficult to differentiate between factual and non-factual information. People have become unsure of what to believe and fear vaccinating their children in case the horrors they see on social media are true. This antivaccine rhetoric has spread from just vaccines to essential prophylactic treatments such as Vitamin K administration in newborns. This is despite ample evidence showing that Vitamin K administration prevents fatal bleeding in newborns. There is also evidence that Vitamin K administration has minimal side effects, demonstrating that the benefits far outweigh the risks. Despite the lack of medical basis, an increasing number of parents are refusing Vitamin K for their newborns. This article explores parents' legal right to make medical decisions for their children, the scope of parental neglect, and whether refusing Vitamin K constitutes neglect from a medicolegal standpoint. It also provides recommendations on addressing this issue.

## Introduction and background

Vitamin K is a fat-soluble vitamin necessary for the synthesis of blood clotting factors involved in coagulation, also known as clotting, within the human body. Clotting is important because it creates a bottleneck over the site of injury to reduce blood loss. This process is also known as hemostasis, or the process of stopping blood flow. Vitamin K is a cofactor, a molecule necessary for an enzyme to function. Enzymes are one of the smallest functional units of the human body, and they facilitate biochemical reactions so that the human body can function properly [1-3]. Vitamin K is a cofactor for the enzyme found in the liver, the gamma-glutamyl carboxylase, which activates several molecules involved in clotting [3]. These molecules, specifically factors II, VII, IX, and X, are needed to reinforce the hemostatic plug, and without them, the plug cannot properly adhere to the site of injury and the injury would continue to bleed through. Vitamin K is necessary to facilitate this process, and without it, the hemostatic plug cannot be reinforced [1-3].

Newborns typically experience a physiological Vitamin K deficiency during the first few weeks of life due to multiple factors contributing to low Vitamin K levels. These include poor placental transfer, low Vitamin K content in breast milk, a sterile gut, and liver immaturity, which leads to inefficient Vitamin K utilization [1,4]. This Vitamin K deficiency is associated with a condition formerly known as hemorrhagic disease of the newborn, now referred to as Vitamin K deficiency bleeding (VKDB) to more accurately reflect its underlying etiology and clinical presentation [5-6]. VKDB is a bleeding disorder that usually manifests in the first few weeks of life after delivery but can manifest up to six months of life [6].

VKDB was first described in medical literature in the late 1800s. In 1894, Boston physician Charles Townsend documented 50 cases of bleeding in newborns. Townsend referred to these cases as *Haemorrhagic Disease of the Newborn*. While he did not know the cause of the bleeding at the time, years later, it was attributed to the inherent Vitamin K deficiency in infants [7]. In 1930, biochemist Henrik Dam discovered that Vitamin K deficiency caused unexpected bleeding in baby chicks [8]. He conducted experiments on chickens fed a low-fat diet and observed prolonged bleeding times. In 1934, Dam found that the addition of hempseed counteracted the bleeding and concluded that the seed contained a substance, Vitamin K, that was needed for blood to coagulate [9]. In 1944, Jörgen Lehmann studied 13,000 infants who were given 0.5 mg of Vitamin K on the first day of life. Lehmann’s research found that infants who received Vitamin K experienced a fivefold reduction in the risk of bleeding to death during the first week of life [8,10]. Finally, in 1961, the American Academy of Pediatrics recommended Vitamin K administration after birth to prevent VKDB [11].

VKDB is characterized by cutaneous bruising or bleeding from mucosal surfaces, the gastrointestinal tract, the umbilicus, the circumcision site, and/or intracranial hemorrhage [12]. VKDB is classified into three types: early-onset, classic, and late-onset. Early-onset VKDB begins within the first 24 hours of age. It usually occurs in mothers taking medications that affect Vitamin K metabolism. These medications include anticonvulsants, antibiotics, antituberculosis agents, and warfarin [6]. These infants may present with a spectrum of diseases, from cutaneous bruising to life-threatening intracranial hemorrhage. Classic VKDB occurs between two days and one week of life. Although some cases occur in infants whose mothers took medications affecting Vitamin K metabolism, most cases are idiopathic, meaning the cause is unknown. Late-onset VKDB occurs between one week and six months of age, with a peak incidence between two and eight weeks. Late-onset VKDB is typically associated with exclusively breastfed infants who did not receive Vitamin K prophylaxis at birth. It may also be associated with liver dysfunction secondary to neonatal hepatitis, bile duct atresia, or intestinal malabsorption. Late-onset VKDB most commonly presents with evidence of intracranial bleeding in 30% to 60% of cases [6]. 

Because newborns have an underdeveloped clotting system, those who do not receive a Vitamin K shot are at a higher risk of VKDB. Early and classic VKDB are more common, occurring in 1 in 60 to 1 in 250 newborns, although the risk is much higher for early VKDB in infants whose mothers used certain medications during pregnancy [13]. Late VKDB is rare, occurring in 1 in 14,000 to 1 in 25,000 infants. Relative to newborns who receive prophylactic Vitamin K, those who do not receive a Vitamin K shot at birth are 81 times more likely to develop late VKDB [13]. Unfortunately, the later the onset, the higher the mortality rate, with late VKDB having a mortality rate of 20-50% [14]. However, with widespread prophylaxis, studies have shown up to a 98% reduction in the incidence of late VKDB [15].

Today, VKDB is rare in the United States, primarily because most newborns receive the Vitamin K shot [15]. Parents generally refuse the Vitamin K shot for several reasons, including concerns about potential harm from foreign substances, a desire for their newborn to remain *natural*, or influence from the opinions of other mothers or family members, whether shared in person or online [16]. However, fears about adverse events from foreign substances are largely unfounded, as actual adverse events are extremely rare. Most reported cases are limited to injection site reactions or anaphylaxis, with one study estimating their occurrence to be as low as 4 in 21 million cases [17].

Approximately 0.25% to 1.7% of newborns who do not receive Vitamin K at birth will experience early or classic Vitamin K deficiency bleeding [18,19]. Before Vitamin K prophylaxis became standard practice, it was difficult to determine the exact number of newborn deaths from VKDB, as infants faced many other complications and diseases before vaccines and other medical advances became widely available [19]. In the early 1800s, before Vitamin K was known to prevent VKDB, only about 57% of children survived to the age of five years. Today, that percentage has increased to about 95% [20]. So while VKDB has not been a common risk for a while, it is now becoming a greater worry for the medical community as more and more parents refuse Vitamin K for their newborns without any sound medical justification.

## Review

Scope of parental rights and how it relates to Vitamin K refusal

Parents have the right to shape their children’s lives, but this right is not unlimited. While they have many rights, they do not have the authority to refuse or deny certain medical treatments for their children. The right of parents to make decisions about their children is a fundamental human right established by the United Nations Convention on the Rights of the Child: “Parents or, as the case may be, legal guardians, have the primary responsibility for the upbringing and development of the child” [21]. States are recommended to respect the primacy of parents, or primary caregivers, as children are physically and emotionally dependent on them for support. Despite the primacy of parents as decision-makers for their children, the Human Rights Committee acknowledges that this right is not absolute, as the state seeks to ensure that parental rights do not impede the child’s right to safety, security, and other fundamental rights [22]. Thus, a balance is needed to ensure that parents' rights are respected while preventing them from impeding the child's right to a full life. 

Court cases have affirmed this balancing act. For instance, *Troxel v. Granville* affirmed the right of parents to direct the care, custody, and control of their children. In this ruling, the Court stated, “Accordingly, so long as a parent adequately cares for his or her children (i.e., is fit), there will normally be no reason for the State to inject itself into the private realm of the family to further question the ability of that parent to make the best decisions concerning the rearing of that parent's children” [23]. This ruling, along with *Washington v. Glucksberg*, 521 U.S. 702 (1997), decided three years before *Troxel v. Granville*, protects parental rights over their children [24].

While parents have the right to raise their children as they see fit, which may include discipline, they, however, do not have the right to neglect their children [25]. Neglect is any act or failure to act by a parent or caretaker that results in death, serious physical or emotional harm, sexual abuse, or exploitation of their child, or any act or failure to act that presents an imminent risk of serious harm to the child. When it comes to state law, neglect is commonly defined as the failure of a parent or another responsible party to provide necessary food, clothing, shelter, medical care, or supervision to the extent that the child’s health, safety, and well-being are at risk of harm. Some states specifically include exceptions for determining neglect, such as religious exemptions for medical neglect and financial considerations for physical neglect [26].

Medical neglect, however, is a hazy area of law. In the broadest definition, neglect can be defined as the failure of a parent or caretaker to provide the resources necessary for the child to grow and thrive [27]. Medical neglect may be considered when a child is brought to medical care too late or when a parent fails to follow what is deemed a reasonable medical plan. While there is no clear overarching definition, neglect can be said to have occurred if a hypothetical *reasonable person* in the same situation would have acted differently. It must be clear that the condition in question poses a significant risk of harm to the child and that the harm could be prevented by accepting effective treatment. The treatment’s benefits must outweigh its potential harmful side effects. Additionally, this safe and effective treatment must be accessible to the patient and caregivers. Finally, the medical provider’s explanations and recommendations must be understood by the family [28]. However, some states explicitly define medical neglect, including New York. The New York Family Court Act defines neglect as “a child less than eighteen years of age whose physical, mental or emotional condition has been impaired or is in imminent danger of becoming impaired as a result of the failure of his parent or other person legally responsible for his care to exercise a minimum degree of care” [29].

In determining whether a behavior constitutes neglect, the *reasonable person* standard can fall short when it comes to religious practice, as reasonable people may disagree over religion. This is particularly relevant in cases involving Jehovah’s Witnesses or Christian Scientists, where parents or primary caregivers may believe that breaking religious rules could jeopardize the child’s immortal soul [28]. The general principle to guide medical neglect can be gleaned from *Prince v. Massachusetts*, “Parents may be free to become martyrs themselves. But it does not follow they are free, in identical circumstances, to make martyrs of their children” [30].

When treatment refusal is determined to constitute neglect, parents or primary caregivers may face criminal liability. However, prosecution rarely occurs. Instead, the courts are asked to exercise their power under the doctrine of parens patriae, which allows state interference to protect a child’s welfare [31]. For example, when a parent or primary caregiver attempts to deny a child chemotherapy for acute lymphocytic leukemia, they are typically met with a court order terminating their custody until the child undergoes the necessary treatment. This is despite chemotherapy having many serious side effects and no guaranteed survival benefit. In the case of cancer treatment, the child’s right to maximize their odds of surviving is more important than the parent’s or primary caregiver’s desire to avoid chemotherapy [20].

The commonality of medical neglect is that it is brought up when there is a clear and present danger to the child, and the parent’s or primary caregiver’s inaction or refusal to follow a treatment standard puts a child in danger or at risk of being put in danger. The case of leukemia and necessary blood transfusions are prime examples of this. Without treatment, a child will most likely die [32]. Therefore, withholding a standard treatment would constitute neglect.

When it comes to Vitamin K refusal, it falls into a more ambiguous area in terms of neglect. A baby who does not receive a Vitamin K shot will not necessarily experience morbidity, unlike a child who is denied chemotherapy or a necessary blood transfusion. Because neglect in this case is more ambiguous, it is more likely to be determined based on the occurrence of morbidity. For example, Pennsylvania, 49 Pa. Code § 47.51 defines any situation that creates “a reasonable likelihood of bodily injury to a child through any recent act or failure to act” as child abuse [33]. This can include failure to secure a child in a car seat, but it is typically not considered relevant unless the unsecured child is injured in a car crash [34]. 

Some state governments have taken the matter into their own hands. For example, the Illinois Department of Children and Family Services (DCFS) Section H states: “...Vitamin K shots or pills to newborns is considered medically necessary. Calls received at SCR concerning a parent or guardian denying consent for the administration of these treatments shall be taken as reports of medical neglect” [35]. Parents alleged that this provision was unconstitutional, and complaints from others led to its repeal in 2018. Despite this, medical professionals in Illinois reported five sets of parents to DCFS in one year for refusing Vitamin K, leading the parents to sue the medical establishments and staff for violations of their Fourth and Fourteenth Amendment rights under 42 U.S.C. §1983 [36]. This case was inconclusive regarding the rights of the parties involved but highlights the clash between parents' rights to determine their children's care and the state's right to intervene for their welfare. 

Vitamin K refusal

In recent years, reports of parents refusing VKDB prophylaxis for their newborns in the United States have been increasing. The reasons why some parents choose not to have their newborn protected from VKDB include their perception of the risk, potential harm from the injection, a desire to be *natural*, a lack of trust in healthcare providers, influences through their social circle, and influences from the internet and/or social media [37,38].

While it is difficult to estimate the rate of refusal as there are no national efforts to record Vitamin K refusal rates, there have been individual studies that attempted to approximate the rate [38,39]. In 2014, the American Academy of Pediatrics looked at studies on Vitamin K refusal in the United States, Canada, and New Zealand and found that the refusal rate showed a small but statistically significant annual increase over the study period, from 0.21% of all live births in 2006 to 0.39% of all live births in 2012 [40]. A recent systematic review from 2020 estimated that refusal rates could be as high as 3.2% of all live births in the United States. With approximately 6 million live births per year in the United States, up to 192,000 newborns could be at risk for VKDB annually [38].

This raises the question: how much avoidable risk to a child is too much to ignore? When it comes to refusing a life-saving blood transfusion or chemotherapy, parents are not allowed to take on the risk of refusal. This makes sense as without the treatment, there is a high likelihood of mortality. However, in the case of Vitamin K refusal, the incidence of VKDB in at-risk newborns without supplementation is significantly lower, ranging from 6% to 12%. However, data on this is limited due to the ethical principles of non-maleficence and beneficence, which make research in this area challenging [41].

The National Highway Traffic Safety Administration estimates that correctly used child restraints reduce fatalities by 71% for infants younger than 1 year old and by 54% for children aged 1 to 4 years old in passenger cars [42]. Is a 6% to 12% reduction in bleeding something that can be ignored? Where is the line drawn?

The fact that there is little data on VKDB raises another issue: we only know a fraction of the morbidity and mortality associated with it. When infants present to a hospital with uncontrolled bleeding after circumcision or umbilical cord separation, or when they exhibit altered mental status due to a hemorrhagic stroke, it is easy to associate these symptoms with VKDB. However, some morbidity may not be discovered until months or years later, as symptoms could manifest when a child is meeting developmental milestones, experiencing learning difficulties in school, or even developing a seizure disorder, all of which could be long-term consequences of micro-hemorrhagic strokes due to Vitamin K deficiency, but are more likely to be associated with genetic disorders or anatomic malformations [39]. Are these risks something that we are okay with as a society?

Recommendation

Policies to prevent VKDB are well-established in the legal system. *Troxel v. Granville* establishes reason for the state to intervene in the private realm of the family when parents fail to adequately care for their children [23]. *Prince v. Massachusetts* establishes that parents cannot martyr their children for their ideologies [30]. Vitamin K supplementation for infants is well-established as medically necessary. It is clear that VKDB can be prevented by accepting effective treatment, namely Vitamin K supplementation. The benefits of the treatment outweigh its potential for harmful side effects. Vitamin K supplementation is also accessible, as it is offered in virtually every mother-baby nursery in the United States. Therefore, it is clear that failure to supplement with Vitamin K would constitute medical neglect. When it comes to medical neglect, the government has the responsibility to exercise its parens patriae role to protect infants at risk from neglect, as their parents have failed them.

## Conclusions

Vitamin K prophylaxis is a simple, safe, and life-saving intervention that has significantly reduced the incidence of VKDB in newborns. However, parental refusal, often influenced by misinformation and distrust in the medical system, creates a public health concern. Ironically, the very success of Vitamin K supplementation in preventing VKDB may contribute to the problem, as many parents remain unaware of its critical role. A concerted governmental effort is needed to track Vitamin K refusal and VKDB cases, whether at the state or national levels. Improved data collection would help quantify the extent of the issue, providing the necessary foundation for legislation aimed at preventing VKDB.

The reluctance to intervene in Vitamin K refusal raises an important ethical question: How much avoidable risk to a child is too much to ignore? In cases like untreated leukemia, the certainty of death without treatment compels authorities to intervene. Yet, when it comes to preventive measures like Vitamin K, the absence of immediate danger should not diminish the obligation to protect vulnerable children. Consider car seat laws - while most car trips do not end in accidents, the potential for harm is significant enough to justify mandatory restraints. If society accepts car seat regulations as a means to safeguard children from preventable harm, why isn't Vitamin K supplementation viewed in a similar way? Addressing these issues requires a multifaceted approach, including provider education, public health initiatives, and potential legal frameworks to ensure newborns receive this essential preventive measure. Moving forward, healthcare professionals, policymakers, and public health organizations must work together to combat misinformation, strengthen advocacy, and reinforce the necessity of Vitamin K administration to safeguard infant health.
